# Brain natriuretic peptide is not predictive of dilated cardiomyopathy in Becker and Duchenne muscular dystrophy patients and carriers

**DOI:** 10.1186/1471-2377-13-88

**Published:** 2013-07-16

**Authors:** Steven Schade van Westrum, Lukas Dekker, Rob de Haan, Erik Endert, Ieke Ginjaar, Marianne de Visser, Anneke van der Kooi

**Affiliations:** 1 Department of Neurology, Academic Medical Centre, University of Amsterdam, Meibergdreef 9, Amsterdam 1100 DD, The Netherlands; 2 Department of Cardiology, Academic Medical Centre, University of Amsterdam, Amsterdam, The Netherlands; 3 Clinical Research Unit, Academic Medical Centre, University of Amsterdam, Amsterdam, The Netherlands; 4 Laboratory of Endocrinology and Radiochemistry, Department of Clinical Chemistry Academic Medical Centre, University of Amsterdam, Amsterdam, The Netherlands; 5 Department of Human Genetics, Leiden University Medical Centre, Leiden, The Netherlands; 6 Current address: Department of Neurology, Martini Hospital, Groningen, The Netherlands; 7 Current address: Department of Cardiology, Catharina Hospital, Eindhoven, The Netherlands

**Keywords:** Duchenne muscular dystrophy, Becker muscular dystrophy, Brain Natriuretic peptide, Dystrophinopathy carriers, Dilated cardiomyopathy

## Abstract

**Background:**

Cardiomyopathy is reported in Duchenne and Becker muscle dystrophy patients and female carriers. Brain Natriuretic peptide (BNP) is a hormone produced mainly by ventricular cardiomyocytes and its production is up regulated in reaction to increased wall stretching. N-terminal-proBNP (NT-proBNP) has been shown to be a robust laboratory parameter to diagnose and monitor cardiac failure, and it may be helpful to screen for asymptomatic left ventricular dysfunction. Therefore we tested whether NT-proBNP can distinguish patients with Duchenne or Becker muscular dystrophy patients and carriers of a dystrophin mutation with a dilated cardiomyopathy from those without.

**Methods:**

In a cohort of Duchenne and Becker muscle dystrophy patients (n = 143) and carriers (n = 219) NT-proBNP was measured, and echocardiography was performed to diagnose dilated cardiomyopathy (DCM).

**Results:**

In total sixty-one patients (17%) fulfilled the criteria for DCM, whereas 283 patients (78%) had an elevated NT-pro BNP. The sensitivity of NT-proBNP for DCM in patients or carriers was 85%, the specificity 23%, area under the ROC-curve = 0.56. In the specified subgroups there was also no association.

**Conclusion:**

Measurement of NT-pro BNP in patients suffering from Duchenne or Becker muscular dystrophy and carriers does not distinguish between those with and without dilated cardiomyopathy.

## Background

It is well known that the heart is involved in Duchenne muscular dystrophy (DMD) and Becker muscular dystrophy (BMD), both caused by mutations in the dystrophin gene on the short arm of the X-chromosome (Xp21). In BMD the dystrophin function is altered in a variable way mostly due to an “in-frame” mutation but nearly always present. In DMD dystrophin is absent (<5%) due to mainly an “out-of-frame”-mutation. Patients with DMD are found to have dilated cardiomyopathy at a median age of onset of 16.8 (range 15–18.7), whereas in BMD median onset is 30.4 (range 23.8-37.0) [1]. Nearly 72% of DMD patients above the age of 18 years have echocardiographic evidence of a dilated left ventricle and a reduced ejection fraction (< 45%) [2]. We and others found a frequency of DCM in BMD patients ranging from 33-49%, which was associated with the duration of the disease [3,4]. In BMD, cardiac involvement can occur in the absence of significant muscle weakness [5]. Carriers of a dystrophin-gene mutation are also at risk of cardiac involvement, often in the absence of muscle weakness [6,7]. In various study populations the incidence of dilated cardiomyopathy (DCM) among DMD carriers varied between 7% to 9%, and among BMD carriers from 0% to 13% [6-8]. Brain Natriuretic peptide (BNP) is a hormone produced mainly by myocytes of the cardiac ventricular wall. Its production is upregulated in reaction to stretching of the wall as occurs in DCM. It is formed by the splicing of proBNP into BNP and the inactive N-terminal proBNP (NT-proBNP) in a 1:1 ratio. Since NT-proBNP is more stable than BNP it is widely used as a marker. In a recent paper about the use of natriuretic peptide (NP) levels, among them BNP and NT-proBNP, in clinical practice Maisel et al. report that the role of NP levels in clinical practice is evolving rapidly and has been incorporated into most national and international cardiovascular guidelines for heart failure [9]. NP levels are found to be quantitative plasma biomarkers of heart failure (HF), are accurate in the diagnosis of HF, can accelerate accurate diagnosis of heart failure presenting in primary care, and may be helpful to screen for asymptomatic left ventricular dysfunction in high-risk patients. In DMD patients raised concentrations of BNP associated with heart failure have also been found [10,11]. Because DCM is considered a severe complication of DMD or BMD and may arise unnoticed in asymptomatic carriers we investigated the prevalence of DCM and the levels of NT-proBNP. A cross-sectional study among patients and carriers was performed in order to address the question whether NT-proBNP might be a marker of the presence of DCM in dystrophinopathies.

## Methods

### Patients

Between January 2002 and February 2005 a convenience sample of patients with DMD, BMD and carriers of these disorders were asked to participate. Since the Clinical Genetic Centre of the University Medical Centre Leiden, the Netherlands, is the referral centre for DNA testing for mutations in the dystrophin gene their database was scrutinized for eligible patients. Subsequently, the treating physicians were asked to contact these patients. Patients were also informed through the patients’ support association (‘Vereniging Spierziekten Nederland’). Inclusion criteria were the following: age 8 to 70 years, and a definite diagnosis of dystrophinopathy. The diagnosis was considered definite when muscle biopsy demonstrated absence of dystrophin on immunohistochemical staining (DMD), or on Western Blot, or when DNA analysis demonstrated the presence of a mutation in the dystrophin gene (DMD, BMD and carriers). Carriers could also be considered definite when pedigree analysis indicated they were obligate carriers or linkage analysis revealed a chance of more than 99% indicating that they were obligate carriers. All were assigned a functional grade based on a scale described by Brooke et al. in order to assess disability due to skeletal muscle involvement [12]. Patients or carriers were considered functionally symptomatic when they experienced disability due to muscle weakness in the upper or lower extremities or both, leading to a Brooke score > 1 (not being able to climb stairs without aid of the railing or worse, and/or abduct the arms in a full circle until they touch above the head or worse). Patients or carriers with DCM without disability due to skeletal muscle weakness were not considered functionally symptomatic. Patients or carriers who recently had had a cardiovascular event, such as myocardial infarction or unstable angina, or who had undergone a major surgical intervention were excluded.

### Investigations

The patients were invited to the out-patient department of the Academic Medical Centre, Amsterdam, the Netherlands. A standardised questionnaire was applied for symptoms suggestive of heart failure such as dyspnoea on exertion, orthopnoea, nocturia, chest pain, palpitations, dizziness or collapse and decrease of appetite. The use of medication was registered. The functional scale designed by Brooke was assessed for both upper and lower extremities [12]. The slow vital capacity, measured with a hand held spirometer, was registered as a percentage of the normal value related to age, height and gender. Blood pressure, height and weight were measured. The Body Surface Area (BSA) and Body Mass Index (BMI) were calculated. Obesity was defined as a BMI > 30.

All patients and carriers were subjected to echocardiography using a Vivid 5 GE echocardiograph equipped with a 5 MHz transducer (LD). Parameters measured were: Left Ventricle End Diastolic Diameter (LVEDD), Left Ventricle End Systolic Diameter (LVESD), both in parasternal long axis projection. Global Left Ventricular Function (LVF) was judged as good, fair, or poor by an experienced cardiologist (L.D.) [13,14]. The Fractional Shortening Index was calculated (FSI) as follows: ((LVEDD-LVESD)/LVEDD)*100%. The LVEDD was corrected for age and body surface area [15]. DCM was defined as an enlarged left ventricle with a global left ventricle dysfunction or fractional shortening of 28% or less, according to guidelines of the World Health Organization/International Society and Federation of Cardiology Task Force [16]. Patients were considered to suffer from chronic heart failure (CHF) when they had symptoms of heart failure, an abnormal ECG and a FSI less than 45%, according tot the guidelines of the task force for the diagnosis and treatment of chronic heart failure of the European Society of Cardiology [17].

Peripheral blood was drawn by venapuncture after at least one-hour rest during the interview for N-terminal pro-brain Natriuretic peptide (NT-proBNP) analysis using Enzyme Immunoassay, Biomedica Medizinprodukte GmbH & CO KG, Vienna, Austria. According to the manufacturer, NT-proBNP levels of less than 350 pmol/L were considered as normal. Independent investigators blinded for the results of the other parameters performed electrocardiography, echocardiography, and laboratory investigations.

### Statistical analysis

Demographic and clinical characteristics of the study group were summarized using descriptive statistics. Differences in proportions, medians and means were analysed using the χ^2^ test, Mann Whitney test, Fisher’s exact test, a two-group t-test or a Mann–Whitney U test, when appropriate. The diagnostic ability of NT-proBNP was expressed in terms of sensitivity, specificity, and area under the ROC curve (AUC). In both the total study group and separate subgroups we analysed the impact of continuous and dichotomous NT-proBNP levels on DCM using multivariate logistic regression, adjusted for disease duration, weakness or complaints suggestive of heart failure. Effect size was expressed in odds ratios (OR).

With a χ^2^-test we additionally analysed the association between an elevated NT-proBNP and the separate parameters of DCM, viz. an enlarged LVEDD, a decreased FSI or global left ventricle hypokinesia at 2D echocardiography. We also compared the level of NT-proBNP in those receiving medication versus those who did not with a χ^2^-test. In addition we analysed the association between diastolic dysfunction and NT-proBNP, DCM and symptoms of heart failure with a χ^2^-test. All analyses were done with SPSS 16.0 for Windows (SPSS Inc. Illinois, USA). When subgroups were compared, they were formed on the basis of their diagnosis (see above). Carriers were further classified by whether they were functionally symptomatic, as described above, or asymptomatic. Statistical uncertainty was expressed in 95% confidence intervals (C.I).

### Informed consent and funding

The study conforms with the principles outlined in the Declaration of Helsinki and had the approval of the Medical Ethical Committee of the Academic Medical Centre, Amsterdam, The Netherlands. All included patients gave informed consent for participation in the study. When an included patient was under the age of 18, informed consent was also given by a parent or guardian.

The work was supported by the Prinses Beatrix Fonds, The Hague, The Netherlands.

The authors are solely responsible for the design and conduct of this study, all study analyses and drafting and editing of the paper.

## Results

The data of 362 out of 404 eligible patients and carriers were available for complete analysis. Thirty-nine patients/carriers were excluded because of missing NT-proBNP data (n = 30), missing echocardiography variables (n = 9) or the body surface area could not be calculated due to deformities (n = 3). A DNA mutation in the dystrophin-gene was present in 71% of the included patients or carriers (85% DMD, 72% BMD and 77% carriers). In the remainder diagnosis was established by immunohistochemical or –biochemical investigation of a muscle biopsy in DMD or BMD patients (13%) or by pedigree or linkage analysis in carriers (16%).

Demographic features and clinical characteristics of the various subgroups are shown in Table 1. Complaints suggestive of cardiac failure such as dyspnea on exertion, orthopnoea, nocturia, chest pain, palpitations were present in 11% of DMD patients, 17% of BMD patients, and 27% of the carriers, respectively. The main symptom, viz. exertional dyspnoea: was found in 2% in DMD 14% in BMD and 13% in carriers. Of the carrier group 93% DMD and 95% BMD carriers were functionally asymptomatic due to the lack of skeletal muscle weakness. ACE inhibitors, β-blockers or diuretics were used by 42 (12%) of patients or carriers. Eight (14%) DMD patients received corticosteroids. None of the patients or carriers had complaints or a history of coronary artery disease. Obesity was present in 3% (5/143) of the DMD or BMD patients and in 16% (35/219) of the carriers (overall 40/362, 11%). Four DMD patients, three BMD patients and none of the carriers (7/362, 2%) had ventilatory support.

**Table 1:** Demographic features and clinical characteristics of 362 DMD/BMD patients and carriers

	**DMD**	**BMD**	**Functionally symptomatic carrier DMD**	**Functionally asymptomatic carrier DMD**	**Functionally symptomatic carrier BMD**	**Functionally asymptomatic carrier BMD**
***n***	55	88	10	130	4	75
**Median age (yr) (range)**	12 (8–44)	30 (8–59)	56 (27–69)	44 (8–68)	47 (22–64)	44 (16–72)
**Median disease duration (yr) (range)**	10 (4–41)	20 (1–52)	10 (0–36)	na	26 (7–44)	na
**Median vital capacity (%) (range)**	52 (14–84)	88 (18–161)	87 (69–125)	106 (61–203)	115 (41–122)	108 (61–166)
**Median diastolic blood pressure (mmHg) (range)**	66 (44–87)	76 (48–112)	82 (67–99)	83 (56–116)	77 (73–90)	77 (60–108)
**Hypertension**^**+**^	2 (4%)	26 (30%)	7 (70%)	54 (42%)	1 (25%)	23 (31%)
**Wheelchair bound**	34 (62%)	12 (14%)	0%	na	0%	na
**Treatment**^**#**^	3 (6%)	17 (19%)	3 (30%)	14 (11%)	1 (25%)	4 (5%)
Any Symptom CHF*	6 (11%)	15 (17%)	7 (70%)	30 (23%)	3 (75%)	19 (25%)
**Obesity**^**§**^	0	5 (6%)	6 (30%)	19 (16%)	1 (25%)	9 (13%)

^#^Treatment: percentage of persons receiving medication including beta-blockers, diuretics, Angiotensin Converting Enzyme-inhibitors (ACE-inhibitors) or Angiotensin II type-1 inhibitors (AT-1-inhibitors).

*Symptoms of CHF: percentage with any symptom of cardiac heart failure (CHF) like nocturia, orthopnea, exercise induced dyspnoea, chest pain or dizziness.

^+^Hypertension: blood pressure higher than 90 mmHg diastolic or 140 mmHg systolic.

^§^Obesity: Body Mass Index >30.

*na* not applicable.

**Figure 1:**
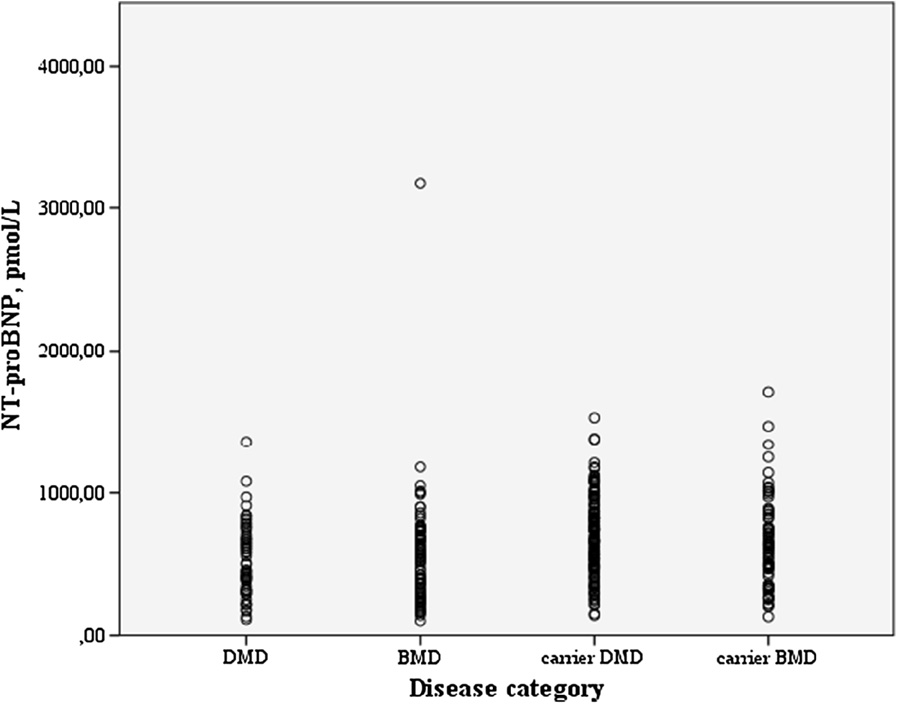
Distribution of concentration NT-proBNP shows no difference amongst the separate disease categories.

In total thirty-three (9%) patients/carriers met the criteria for chronic heart failure. The echocardiographic variables are shown in Table 2. In total 283 patients/carriers (78%) had an elevated NT-pro BNP. The point-prevalences of patients or carriers fulfilling the defined criteria for *chronic* heart failure (CHF) or DCM in the DMD patients were 6% and 16% (3/55 and 9/55), respectively, in the BMD patients 14% and 32% (12/88 and 28/88), respectively and in the carriers 8% and 11% (18/219 and 24/219), respectively. Overall, DCM was present in 61 out of 362, 17%. No significant difference in the frequencies of the presence of DCM between the groups with and without an elevated NT-proBNP was found (52/283 vs. 9/79 i.e.:18% vs. 11%, *p* 0.14) (Table 3). There was also no significant difference in the frequencies of chronic heart failure between the groups with and without an elevated NT-proBNP (11% vs 9%, *p* 0.06 Chi-square). The distribution of the NT-proBNP concentration among the different disease categories is shown in Figure 1. Only in DMD patients the median NT-proBNP level is significantly higher among patients with DCM (440 vs. 840 pmol/L, *p* 0.01) (Table 4).

**Table 2:** Echocardiographic variables

	**DMD**	**BMD**	**Functionally symptomatic carrier DMD**	**Functionally asymptomatic carrier DMD**	**Functionally symptomatic carrier BMD**	**Functionally asymptomatic carrier BMD**
LVEDD (mm, range)	42 (35–66)	54 (33–77)	52 (43–64)	50 (40–69)	51.5 (42–58)	49 (39–78)
FSI (%, range)	33 (5–48)	30 (3–48)	35 (14–48)	33 (13–55)	32 (24–40)	34 (5–52)
LVF Dysfunction^§^	20%	34%	14%	10%	0%	3%

*LVEDD* Left Ventricle End Diastolic Diameter, *FSI* Factional shortening Index, *LVF* Left Ventricular Function.

§ LVF dysfunction is any function less than good.

**Table 3:** Elevated NT-proBNP in relation to the presence of dilated cardiomyopathy

		**DCM**		
		**Present**	**Absent**	
NT-proBNP	Elevated	52	231	283
	Normal	9	70	79
		61	301	362

**Table 4:** Median concentration NT-proBNP in pmol/L per disease category

	**Dilated cardiomyopathy**		
	**Absent**	**Present**	**p**^**§**^
DMD (range)	440 (110–840)	840 (170–1360)	0.01
BMD median (range)	510 (100–1180)	580 (160–3170)	ns
Carrier DMD (range)	660 (140–1530)	665 (400–1380)	ns
Carrier BMD (range)	600 (130–1710)	845 (350–1340)	ns
Carrier overall (range)	640 (130–1710)	665 (350–1380)	ns

§Mann–Whitney test.

**Figure 2:**
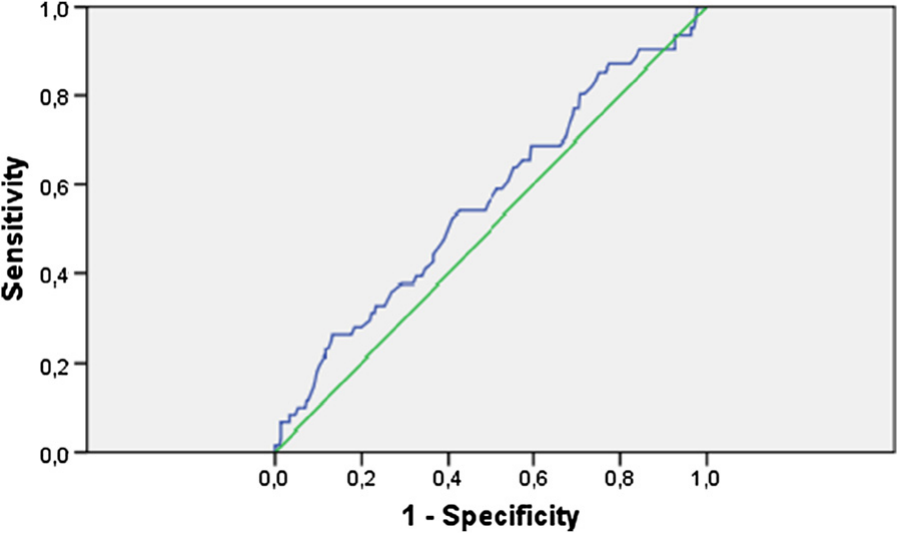
Receiver Operating Curve of NT-proBNP for DCM in the whole cohort.

The sensitivity (52/61) rate and specificity (70/301) rate of NT-proBNP for the presence of DCM in the whole cohort were 85% and 23%, respectively. The area under the ROC curve (AUC) was 0.56 (95% C.I.: 0.48-0.65) (Figure 2). The sensitivity (30/33) and specificity (76/329) rates of NT-proBNP for the presence of chronic heart failure were 91% and 23%, respectively. The AUC was 0.58 (95% C.I.: 0.49 – 0.68).

No significant association between elevated NT-proBNP and DCM was found in the subgroups, i.e., DMD, BMD, asymptomatic or symptomatic carriers (data not shown) When NT-proBNP level was entered as a dichotomous independent variable (elevated or not) into a logistic regression model (adjusted for disease duration, weakness, treatment or complaints suggestive of heart failure) no statistical significant associations could be demonstrated. In DMD patients: OR = 2.16 (95% C.I. 0.21-22.79, *p* 0.52); in BMD patients: OR = 0.92 (95% C.I. 0.30 -2.83, *p* 0.89); in carriers: OR = 4.84 (95% C.I. 0.51-31.58, *p* 0.19). In the total study group the OR was 1.49 (95% C.I. 0.67-3.34, *p* 0.33). When NT-proBNP concentration was entered as a continuous variable into the model, the parameter did not explain the presence of DCM in the total study group (OR = 1.87, 95% C.I. 0.68-5.13, *p* 0.23). Also, NT-proBNP was not associated with the presence of DCM in BMD patients separately (OR = 1.9 (95% C.I. 0.40-9.11, *p* 0.42) or carriers (OR = 2.26, 95% C.I. 0.40-9.75, *p* 0.40). In DMD patients the regression model could not be fitted statistically because of the low incidence of DCM in this subgroup.

Neither could we demonstrate a significant difference in the frequencies of an elevated NT-pro BNP between patients or carriers with and without an LVEDD exceeding the maximal LVEDD corrected for age and body surface area (82% vs. 76%) (*p* 0.22), with or without a fractional shortening index less than 28% (82% vs. 77%, *p* 0.25) or with or without global left ventricle hypokinesia at 2D echocardiography (85% vs. 77%, *p* 0.22).

The frequency of an elevated (> 350 pmol/L) NT-proBNP among patients or carriers receiving medication was 39 (93%) compared to 244 (76%) among those without medication (p = 0.01). The level of NT-proBNP was significantly higher in those receiving medication compared to those without (800 vs 590 pmol/L, p < 0.001).

## Discussion and conclusions

In this cross-sectional study encompassing a large number of DMD and BMD patients and carriers the overall point prevalence of dilated cardiomyopathy was 17%. Across the whole group, there was no significant difference in the frequency of elevated NT-proBNP levels between individuals with or without dilated cardiomyopathy. There was no relation between an elevated NT-proBNP and DCM when corrected for disease duration, treatment any weakness or complaints of heart failure. Only in DMD patients with DCM the median NT-proBNP level was higher than in DMD patients without DCM. However, it was impossible to statistically correct for disease duration, treatment, any weakness or complaints of heart failure with a logistic regression analysis since the model did not fit. The ROC- analysis did not show a more discriminatory threshold. Our goal was to assess whether NT-proBNP might be a marker of the presence of DCM in dystrophinopathies and the study showed that the overall accuracy of the NT-proBNP test to diagnose dilated cardiomyopathy was poor.

It is of note, that complaints suggestive of cardiac failure, in particular exercise-induced dyspnoea, were most often found in the carriers of dystrophin mutations (all carriers: 59/219, 27%) as compared to patients with DMD (11%) or BMD (17%). There may be several explanations for this counterintuitive observation. First, there is no consensus about the validity and reliability of symptoms indicating chronic heart failure [17]. Second, the differences in the frequency might be explained by the interaction between physical capabilities and the severity of the loss of function of dystrophin and the inverse relationship between these two elements. None of the carriers were wheelchair bound so the cohort of carriers seems to be more capable of performing exercise-related activities than BMD and DMD patients of whom 14% and 62% respectively were wheelchair bound. In line with that BMD patients who have intermediate function of dystrophin as compared to DMD and carriers have more complaints of heart failure than do DMD patients. Third, muscle weakness might lead to higher demands on the cardiac system in patients who remain ambulatory and consequently leads earlier to complaints of heart failure. Fourth, the higher frequency of obesity in the cohort of carriers (16% in carriers vs. 3% in patients) is a confounding factor. However, the low percentage of obesity found in patients could be an underestimation because patients have a variable level of muscle atrophy which diminishes the total body mass.

The frequency of DCM was 11% in carriers, as compared to 16% in DMD and 32% in BMD. Others have found prevalences up to nearly 72% of DMD patients above the age of 18 years [2]. The relatively low prevalence of DCM in our DMD patients may be explained by the fact that 80% of the patients was below the age of 20, and by our strict definition of DCM where others used less stringent criteria, i.e., echocardiographic evidence of a dilated left ventricle and a reduced ejection fraction (< 45%) [2].

BNP has been previously studied as marker for left ventricle dysfunction in patients with Duchenne and Becker muscular dystrophy and in carriers [10,18-20]. Mori et al. showed a curvilinear relationship between BNP and the shortening fraction of the left ventricle in 63 DMD patients [10]. When the shortening fraction was found to be below 15% the BNP level rose exponentially. Thereupon the authors concluded that BNP was a marker for a severely affected ejection fraction, but not a sensitive marker for early left ventricle dysfunction. This was confirmed by Demachi et al. and Mohyuddin et al. [19,21]. Demachi compared 31 patients with DMD/BMD with 20 patients with idiopathic dilated cardiomyopathy. The former had lower levels of BNP than the latter, especially when the left ventricle ejection fraction was markedly diminished. They hypothesised that the difference may well be explained by the lesser mechanical strain of the left ventricular myocytes and epicardial fibrosis in DMD/BMD and the reduced physical activity since all patients in the study were wheelchair dependent. A linear correlation between BNP and fractional shortening or left ventricle diameter was found in 15 DMD carriers described by Adachi [20]. In 65% of the carriers with an enlarged left ventricle and in 50% of the carriers with decreased fractional shortening an elevated BNP was present.

In contrast to the afore-mentioned publications we adhered to a strict definition of DCM according to the guidelines of the World Health Organisation/International Society and Federation of Cardiology Task Force, namely an enlarged LVEDD corrected for weight and age together with a global left ventricle dysfunction or a fractional shortening index (FSI) of less than 28%, according to guidelines of the World Health Organisation/International Society and Federation of Cardiology Task Force [16]. The relative low incidence of DCM might explain why NT-proBNP failed to discriminate between DCM negative and DCM positive patients/carriers when corrected for age, weakness, treatment and complaints in a logistic regression analysis.

Treatment of left ventricle dysfunction in muscular dystrophies is studied in DMD patients, not in carriers. In DMD patients dysfunction of the left ventricle function can be influenced by treatment [22-25]. In trials on patients with acute or chronic heart failure on the basis of other diseases than a dystrophinopathy, it has been shown that NT-proBNP decreases if treatment of heart failure due to left ventricle dysfunction is successful [26,27]. Therefore it might be expected that treatment with ACE-inhibitors with or without beta-blockers irrespective of the indication of treatment in DMD or BMD can also result in a decreased level of natriuretic peptides. In the Netherlands dystrophinopathy patients and carriers tend to be treated since the frequency of treated patients or carriers was significantly higher in those with DCM compared to those without DCM. However, it seems unlikely that treatment has interfered with the diagnostic ability of NT-proBNP for DCM since patients or carriers on this medication had more often an elevated NT-proBNP and a higher mean NT-proBNP than those without DCM.

NT-proBNP was found to be significantly correlated with depressed LV function on echocardiography in a cohort of 28 wheelchair dependent DMD patients who were on a (non)invasive mechanical ventilator [11]. In contrast, our cohort was more heterogeneous showing differences in disease severity (2% on mechanical ventilation and 13% wheelchair dependent), age and co-morbidity. However, neither in the whole cohort nor in the separate subgroups an elevated NT-proBNP was associated with DCM. Given the relatively high sensitivity of NT-proBNP for DCM it might be considered as a screening tool but compared to the non-invasive echocardiogram, NT-proBNP does not outperform the echocardiogram as a screening tool. The heterogeneity of the cohort, the lack of association when adjusted for treatment, weakness or disease duration strengthen the conclusion that measurement of NT-pro BNP in patients suffering from Duchenne or Becker muscular dystrophy or carriers is not helpful to diagnose dilated cardiomyopathy in any phase of the disease.

## Competing interests

The authors declare that they have no competing interests.

## Authors’ contributions

SSvW: made a substantive intellectual contribution to the submitted manuscript: design and conceptualization of the study, analysis and interpretation of the data, and drafting and revising the manuscript. LD: author, made a substantive intellectual contribution to the submitted manuscript: conceptualization of the study, interpretation of the data, and drafting and revising the manuscript. RdH: author, made a substantive intellectual contribution to the submitted manuscript: design of the study, analysis of the data, and drafting and revising the manuscript. EE: contributor. IG: contributor. MdV: author, made a substantive intellectual contribution to the submitted manuscript: design and conceptualization of the study, interpretation of the data, and revising the manuscript. AvdK: made a substantive intellectual contribution to the submitted manuscript: design and conceptualization of the study, interpretation of the data, and drafting and revising the manuscript. All authors read and approved the final manuscript.

## Pre-publication history

The pre-publication history for this paper can be accessed here:

http://www.biomedcentral.com/1471-2377/13/88/prepub
